# Dissection of *C. elegans* behavioral genetics in 3-D environments

**DOI:** 10.1038/srep09564

**Published:** 2015-05-08

**Authors:** Namseop Kwon, Ara B. Hwang, Young-Jai You, Seung-Jae V. Lee, Jung Ho Je

**Affiliations:** 1 X-ray Imaging Center; 2 School of Interdisciplinary Bioscience and Bioengineering; 3 Department of Life Sciences; 4 Division of Information Technology Convergence Engineering,; 5 Department of Materials Science and Engineering, Pohang University of Science and Technology, Pohang, South Korea; 6 Department of Biochemistry and Molecular Biology, Virginia Commonwealth University, Richmond, Virginia, USA

## Abstract

The nematode *Caenorhabditis elegans* is a widely used model for genetic dissection of animal behaviors. Despite extensive technical advances in imaging methods, it remains challenging to visualize and quantify *C. elegans* behaviors in three-dimensional (3-D) natural environments. Here we developed an innovative 3-D imaging method that enables quantification of *C. elegans* behavior in 3-D environments. Furthermore, for the first time, we characterized 3-D-specific behavioral phenotypes of mutant worms that have defects in head movement or mechanosensation. This approach allowed us to reveal previously unknown functions of genes in behavioral regulation. We expect that our 3-D imaging method will facilitate new investigations into genetic basis of animal behaviors in natural 3-D environments.

For genetic dissection of animal behaviors, *C. elegans* has been used widely as a model, largely due to its powerful genetics and fully characterized simple nervous system^1^. Despite extensive analysis, behavioral studies of the worm have been performed under two-dimensional (2-D) conditions, such as on surfaces of agar plates^2^ or in microfluidic chips^3^, because of the 2-D limitation of conventional imaging methods. The 2-D conditions are far from the worm’s natural habitats such as soil or rotting fruits that are essentially 3-D environments^4^. Understanding of the animal behaviors under 3-D environments is thus necessary for finding out the natural functions of genes.

X-ray microtomography^5^ or laser diffraction analysis^6^, reported for quantification of the worm movement in 3-D environments, is not appropriate for behavioral study of the worm. Scanning time is extremely slow (~80 minutes) in X-ray microtomography and merely one parameter (oscillation frequency) is affordable in laser diffraction analysis. A dual-view imaging method, known as 3-D Worm Tracker (3DWT), recently developed for quantification of *C. elegans* movement in 3-D environments^7^, had two major limitations. First, occluded body parts cannot be reconstructed. Secondly, its labor-intensive manual 3-D tracking restricts providing sufficient data sets for quantification.

In this study, we proposed 3-D Worm Tracker (3DWT) 2.0 for behavioral analysis of *C. elegans* in 3-D environments. We invented a multi (triple)-view imaging system that enables not only automatic 3-D tracking but also 3-D reconstruction from multiple images including occlusion of body parts. Using the 3DWT 2.0, we quantified behavioral phenotypes of mutant worms in 3-D environments, by using head movement-defective or mechanosensory-defective mutant worms. We uncovered novel roles of the head muscular system and mechanosensory pathways of the worm in 3-D behavioral regulation. The 3DWT 2.0 has a potential for applications to analysis of various worm behaviors to elucidate genetic basis of natural animal behaviors.

## Results

### Development of 3-D worm tracker 2.0

**Figure 1:**
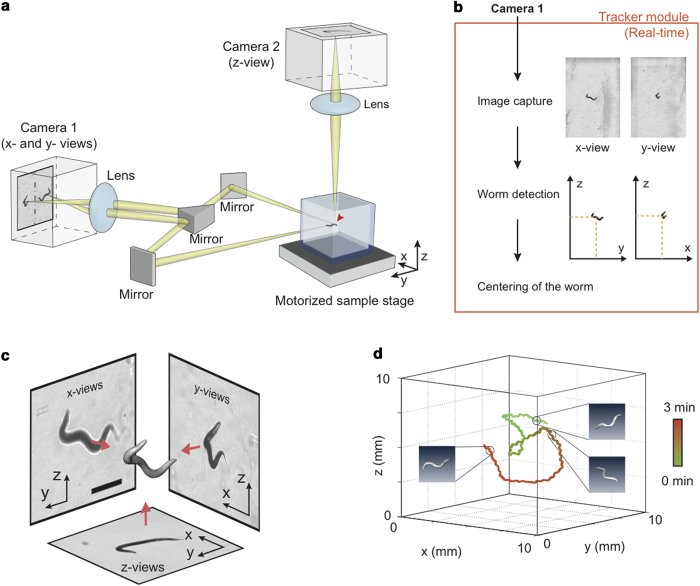
Worm Tracker 2.0. 3-D (**a**) Multi (triple)-view imaging system for 3-D worm tracker 2.0. A red arrowhead indicates the focal point. (**b**) Automatic 3-D tracking process of a single worm. (**c**) Reconstruction of a 3-D posture of a worm based on back-projections of x-, y- and z-views (scale bar represents 300 μm). (**d**) A trace and three representative reconstructed images of a worm moving in a gelatin cube (>2 cm^3^) for 3 minutes.

The apparatus of the 3-D Worm Tracker (3DWT) 2.0 is based on a multi (triple)-view imaging system that simultaneously provides three perpendicular x-, y-, and z-views (Fig. 1a). Our novel system is designed to capture the x- and y-views simultaneously by the camera 1 and the z-view by the camera 2 with the same focal point (red arrowhead). The stereoscopic imaging of x- and y-views on each half of the sensor of camera 1, which efficiently enables automatic 3-D tracking, is achieved by using a set of mirrors that steer the lights from the x- and y-directions of the sample into the camera 1. For automatic 3-D tracking, we programmed a tracker module, a custom software that analyzes the position of a worm from the captured x- and y-views (Fig. 1b). The module also controls a motorized stage to locate the worm at the focal point every 1.4 s (time interval), thereby tracking the worm in real-time (Fig. 1b and see Methods for details).

We also programmed an analyzer module (See Methods) for 3-D posture and 3-D trajectory of the worm. A back-projection algorithm^8^ was employed for the reconstruction of each 3-D posture of the worm from each set of its three (x-, y-, and z-) views, taken by the 3DWT 2.0 (Fig. 1c and see Methods for details). Figure 1c illustrates the successful reconstruction of a complex 3-D posture of a worm, overcoming the occlusion limitation of the previous 3DWT^7^. In addition, the back-projection algorithm greatly simplified the reconstruction process by stripping out additional image processing such as skeletonization and stereomatching of the previous 3DWT. To evaluate the computational error of the reconstruction, we calculated the coefficient of variation of the reconstructed volumes and confirmed that the value was as small as 10%, which is comparable to 8.9% obtained from the cylinder approximation method for 2-D worm images^9^. The analyzer module also allowed us to quantitatively measure the trajectory of the worm (Fig. 1d and Supplementary Movie 1) and its locomotory parameters such as speed and reorientations automatically. Thus, 3DWT 2.0 enables comprehensive behavioral analysis of *C. elegans* moving in 3-D environments.

### Locomotory behaviors of wild-type *C. elegans*

Using the 3DWT 2.0, we examined locomotory behaviors of wild-type worms crawling in 2-D and 3-D environments by using thin films (100 μm thickness) and cubes (>2 cm^3^) of gelatin, respectively (Supplementary Fig. 1 and see Methods for details). From the trajectories of the worms, we measured their i) speed, ii) reorientation frequency, and iii) curving rate (See Methods).

**Figure 2:**
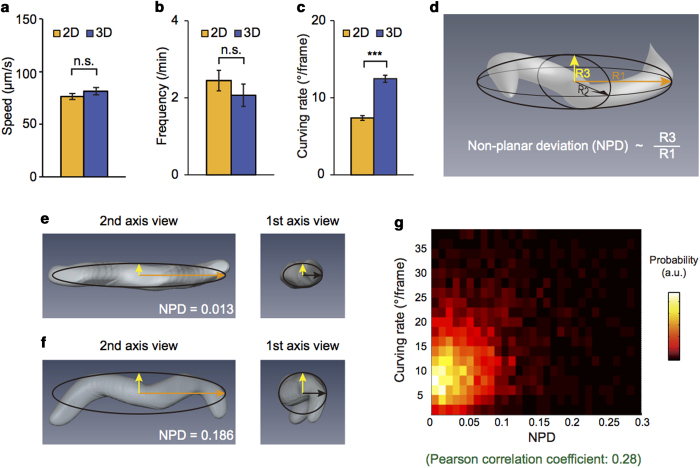
Locomotory behaviors of wild-type worms. (**a-c**) Average speeds (**a**), reorientation frequencies (**b**) and curving rates for forward runs (**c**) of wild-type worms in 2-D and 3-D environments. Error bars represent SEM. n.s. (not significant), ***p *<* *0.0005,* Mann-Whitney U-test. (**d**) Schematic for a best-fit ellipsoid of a 3-D postures of a worm. The orange, black and yellow axes represent three semi-principal axes with lengths R1, R2 and R3, respectively. Non-planar deviation (NPD) is defined as a value proportional to the ratio of R3 to R1. (**e**-**f**) Two representative “planar” (**e**) and “non-planar” (**f**) postures of worms with best-fit ellipsoids. The images were captured in two views: from the second and the first axes. (**g**) Relationship between NPDs and curving rates. The colors represent probability of the distribution. The curving rate has a positive correlation with normalized deviation as the Pearson correlation coefficient (0.28) indicates. The data were obtained by measuring the crawling locomotion of wild-type worms in 2-D and 3-D environments (n = 38 for 2-D and n = 35 for 3-D experiments).

We found that the crawling speeds of wild-type animals were similar in 2-D (mean ± SEM: 76 ± 3 μm/s, n = 38) and 3-D (81 ± 4 μm/s, n = 35) environments (Fig. 2a). The reorientation frequency, counted for curving rates of >135°/frame (See Methods)^10^, slightly decreased in 3-D environments compared to that in 2-D environments, but the decrease was not significant (Fig. 2b). Together these results indicate that worms navigate 2-D and 3-D environments with similar locomotory patterns in speed and reorientation, two important parameters in the worm behaviors^11^,^12^.

However, curving rates during forward runs (<60°/frame) (See Methods) showed a significant difference between 2-D and 3-D environments. Specifically, the average curving rates largely increased from 7.4 ± 0.3°/frame in 2-D to 12.5 ± 0.5°/frame in 3-D environment (Fig. 2c). The distribution of the curving rates, which had a maximum near 0°/frame in 2-D environments, exhibited a peak shift to ~5°/frame in 3-D (Supplementary Fig. 2a). Together, these results indicate that the degree of curving is larger in 3-D than in 2-D environments.

We show that the higher curving rate under 3-D environments is due to the increased degrees of freedom of the worm in 3-D environments. For this, we analyzed the posture of the worm, which reflects the degree of freedom of movements. The posture can be represented by a best-fit ellipsoid with the semi-principal axes of length, R1, R2, and R3 (R1 > R2 > R3), calculated with principle component analysis (PCA) (Fig. 2d) (See Methods). How much the posture deviates from a plane can be quantitatively described by ‘Non-Planar Deviation’ (NPD), defined as
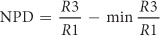
where the second term is the least of the ratios of R3 to R1 (Supplementary Fig. 3). NPD well represents the complexity of the posture, as demonstrated by a ‘planar’ (NPD = 0.013) and a ‘non-planar’ (NPD = 0.186) postures in Fig. 2e,f, respectively. In fact, the postures of wild-type worms crawling in 3-D environments showed a wide NPD distribution, mostly within 0 and 0.3 (99%) (Supplementary Fig. 4). The wide NPD distribution clearly indicates that degrees of freedom of the worm increase in 3-D environments. Interestingly, the NPD was positively correlated with the curving rate for forward runs in 3-D environments (Pearson correlation coefficient: 0.28) (Fig. 2g). This result suggests that the increased curving in 3-D environments (Fig. 2c) is due to the additional degrees of freedom of worms, which are physically restrained in 2-D environments.

### Contributions of the head movements to 3-D locomotion

The head of *C. elegans* can move in all directions, but the other body part can move only in dorsoventral directions^13^. We therefore hypothesized that the head muscular system plays important roles in the 3-D locomotion of *C. elegans*. To test this hypothesis, we examined the locomotion of two mutants that are defective in normal head movements: *vab-10(e698)* (spectraplakin) mutants that display a feeble head phenotype due to head muscle dystrophy^14^,^15^ and *eat-4(n2474)* (vesicular glutamate transporter) mutants that show excessively frequent head lifts^16^.

**Figure 3:**
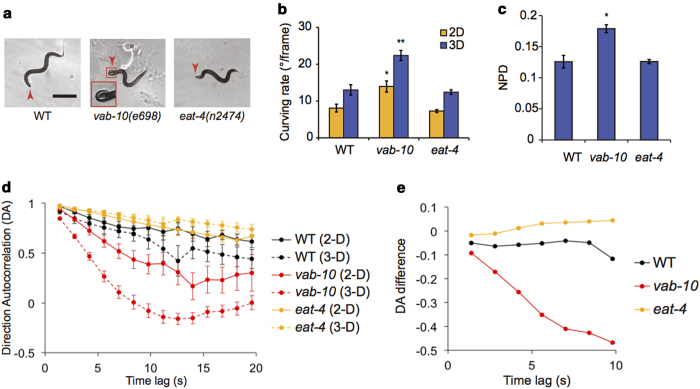
Locomotory behaviors of mutants with abnormal head movements. (**a**) Representative images of a wild-type, a *vab-10(e698)* mutant, and an *eat-4(n2474)* mutant animals in 3-D environments (the scale bar represents 400 μm). Arrowheads indicate the heads of the worms and the inset is a magnified image of the bent head of a *vab-10* mutant (**b**) Curving rates for forward runs of wild-type and the mutant animals in 2-D and 3-D environments. (**c**) The average values of the non-planar deviation (NPD) of reconstructed 3-D postures. *p *<* *0.005*, **p *<* *0.001,* Mann-Whitney U-test. (**d**) Direction Autocorrelations (DAs), which represent directional changes during given time lags. (**e**) Differences between the DAs in 2-D and 3-D environments. Because *vab-10* mutants did not dig into the solidified 3% gelatin, wild-type, *vab-10* mutant, and *eat-4* mutant animals were loaded into 2% (w/v) gelatin solution at 20 °C, and habituated for 40 minutes for both 2-D and 3-D experiments in Fig. 3. We examined the following numbers of worms: n = 13 and 8 for wild-type animals; n = 8 and 14 for *vab-10* mutants; n = 16 and 14 for *eat-4* mutants for 2-D and 3-D experiments, respectively. Error bars represent SEM.

We first confirmed that *vab-10* mutants displayed a feeble head phenotype. Specifically, the *vab-10* mutants had little movement in their heads (see Supplementary Movie 2) and occasionally displayed a bent-head phenotype (red arrowheads in Fig. 3a). Notably, the curving rate for forward runs was significantly higher in *vab-10* mutants (22.4 ± 1.4°/frame) than that in wild type (13.0 ± 1.4°/frame) under 3-D environments (Fig. 3b). We noted that the curving rate of *vab-10* mutants (14.0 ± 1.5°/frame) in 2-D environments is also larger than that of wild-type worm (8.1 ± 1.1°/frame) but the difference was much larger in 3-D environments (Fig. 3b). In addition, we found that the non-planar deviation (NPD) of *vab-10* mutants was larger than that of wild type (Fig. 3c). The mutation in *vab-10* gene also impaired the overall locomotion of *C. elegans* in 3-D environments (Supplementary Fig. S5). Together, these results imply that well-controlled 3-D locomotion requires normal head muscular system. Different from *vab-10* mutants, *eat-4* mutants showed no abnormality in curving rate and NPD in either 2-D or 3-D environments (Fig. 3b,c). This result indicates that the 3-D locomotion, as quantified here by the two parameters, is regulated independently of EAT-4.

Directional change is a fundamental parameter to understand the behavioral strategies of *C. elegans*^17^. The directional change can be quantified by Direction Autocorrelation (DA), which represents similarities between moving directions of a single worm as a function of the time lag between the moving directions (see Methods for detail)^18^. Thus, if the moving directions with a time lag are the same or the opposite, the DA will be +1 or −1, respectively. If the moving directions lose a correlation, the DA will be near zero. We found that the DA for a short time lag (1.4 s) was near +1 (0.97 and 0.92 for wild type, 0.94 and 0.85 for *vab-10*, and 0.97 and 0.96 for *eat-4* in 2-D and 3-D environments, respectively), indicating the directional change for a short time lag was small (Fig. 3d). However, the directional change increased over a long time lag, as DA decreased. Remarkably, *vab-10* mutants displayed a fast decrease in DAs compared to wild type (Fig. 3d), indicating that *vab-10* mutants have defects in maintaining their moving directions. The differences in DAs, specifically the subtraction of DA in 2-D from that in 3-D conditions, were very small (~near zero) in both wild type (−0.06 ± 0.01) and *eat-4* mutants (0.02 ± 0.01) (Fig. 3e). This implies that the directionality of wild type and *eat-4* mutants is largely unaffected by the increased degrees of freedom of movements in 3-D environments. In *vab-10* mutants, however, the DA difference significantly decreased with time lag, indicating that the impairment in the directionality caused by *vab-10* mutations become more severe in 3-D than in 2-D environments.

### Role of mechanotransduction channels in 3-D locomotion

*C. elegans* modulates their locomotory behaviors through sensing physical surroundings by mechanosensory systems^11^,^19^. Do mechanosensory systems modulate the worm behaviors differently in 3-D environments? To answer this question, we investigated locomotory phenotypes of mechanosensory-defective mutants in 2-D and 3-D environments. These include *mec-4(e1339)* (DEG/ENaC) and *mec-10(e1515)* (DEG/ENaC) mutants, which are defective in gentle body touch responses^20^,^21^. In addition, we examined *osm-9(ky10)* (TRPV) and *trpa-1(ok999)* (TRPA) mutants, which are defective in head touch responses^22^,^23^, and *trp-4(sy695)* (TRPN) mutants, which are defective in stretch sensation^24^.

**Figure 4:**
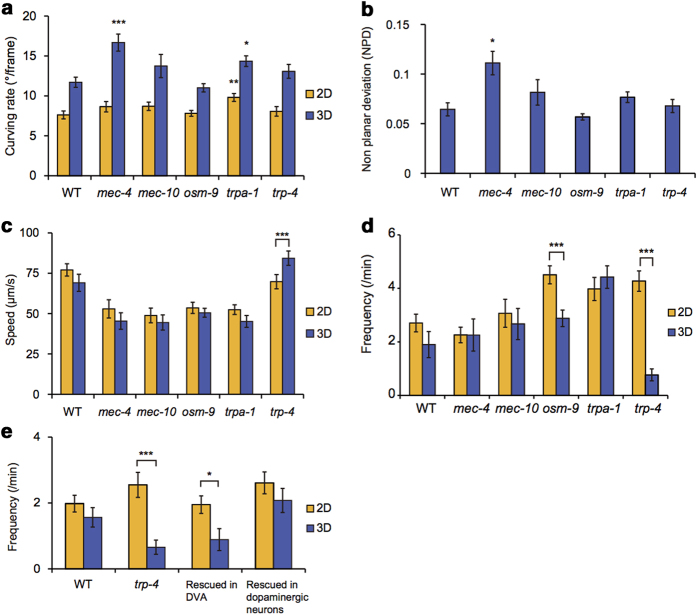
Locomotory behaviors of mechanosensary defective mutants. (**a-d**) Curving rates for forward runs (**a**), non-planar deviations (NPDs) of 3-D posture (**b**), speeds (**c**), and reorientation frequencies (**d**) of indicated strains (n = 18 and 14 for wild-type; n = 17 and 14 for *mec-4(e1339)*; n = 15 and 11 for *mec-10(e1515)*; n = 20 and 24 for *osm-9(ky10)*; n = 26 and 30 for *trpa-1(ok999)*; and n = 16 and 11 for *trp-4(sy695)* mutants for 2-D and 3-D experiments, respectively). The reorientation frequencies^10^ were significantly reduced in 3-D compared to those in 2-D for *osm-9* and *trp-4* mutants while showing little differences for wild type and *mec-4*, *mec-10* and *trpa-1* mutants. In addition, we found that *ocr-2(ak47)* and *glr-1(n2461)* mutants, which are defective in OSM-9-mediated mechanosensation^31^,^26^, also showed reduced reorientation frequencies in 3-D environments (Supplementary Fig. 6). (**e**) Reorientation frequencies of wild-type, *trp-4(sy695)* and *trp-4(sy695)* mutant animals expressing *trp-4* in DVA neuron driven by a *twk-16* promoter or in dopaminergic neurons driven by a *dat-1* promoter^24^ (n = 9 and 16 for wild-type; n = 26 and 21 for *trp-4* mutants; n = 25 and 28 for *trp-4(sy695); Ptwk-16(DVA)::trp-4;* and n = 27 and 25 for *trp-4(sy695); Pdat-1::trp-4* for 2-D and 3-D experiments, respectively). Statistical analysis was performed to compare mutants with wild-type animals (**a-b**) or between 2-D and 3-D data (**c-e**) (*p *<* *0.005*, **p *<* *0.001*, ***p *<* *0.0005*, Mann-Whitney U-test). Error bars represent SEM.

Compared to wild type, *mec-4* mutants showed high curving rates in 3-D conditions but little difference in 2-D conditions (Fig. 4a). In addition, the non-planar deviation (NPD) of *mec-4* mutants was significantly larger than that of wild type (Fig. 4b). Together these results imply that DEG/ENaC MEC-4 plays crucial roles in regulating 3-D locomotion, in terms of curving and 3-D posture. On the other hand, *trpa-1* mutants showed high curving rates in both 2-D and 3-D environments (Fig. 4a), while displaying no significant difference in NPD (Fig. 4b), compared to wild type. This suggests that the locomotory phenotype of *trpa-1* mutants is independent of the spatial dimension of physical environments.

The locomotion speeds of wild type and *mec-4*, *mec-10*, *osm-9* and *trpa-1* mutants showed no significant differences regardless of the spatial dimensions (Fig. 4c). In contrast, *trp-4* mutants displayed an increased moving speed in 3-D conditions compared to 2-D. *trp-4* mutants also showed significantly reduced reorientation frequency in 3-D environments (Fig. 4d). These results indicate that TRP-4/TRPN mechanotransduction channel plays different roles in 3-D than in 2-D environments.

To understand the TRP-4-mediated regulation of reorientation at the cellular level, we asked in which cell(s) the TRP-4 channels functioned in regulation of the reorientation. TRP-4 channels are expressed in interneurons including DVA neuron and in the CEP and ADE dopaminergic neurons^25^. Thus, we used transgenic animals that expressed trp-4 specifically in DVA interneuron or dopaminergic neurons^24^ examined whether the transgene rescued the *trp-4* mutant phenotype. We found that dopaminergic neuron-specific *trp-4* expression restored the reorientation phenotype of *trp-4* mutants in 3-D environments, whereas DVA-specific *trp-4* expression did not (Fig. 4e). This result indicates that TRP-4 in dopaminergic neurons regulates the reorientation of worms in 3-D environments.

## Discussion

For *C. elegans* behavioral genetics, here we developed a novel three-dimensional worm tracker (3DWT 2.0) that allowed us to successfully reconstruct all the 3-D postures of crawling worms based on a multi (triple)-view imaging. In particular, automatic 3-D tracking in 3DWT 2.0 enabled us to analyze thousands of 3-D postures and positions with less labor. We for the first time discovered the positive correlations between curving rates and 3-D postures (Fig. 2g), thanks to the simultaneous recording of both 3-D trajectories and 3-D postures.

In contrast to the body of *C. elegans*, its head is known to move in all directions. *vab-10* mutants that are incapable of moving head, however, showed exaggerated 3-D locomotion (Fig. 3b,c). This suggests that the 3-D locomotion, as quantified here, can be generated by the other body parts or external forces from physical surroundings. However, the additional loss of directionality of the mutant worms (Fig. 3e) indicates the importance of head in navigation behaviors of the worm in natural 3-D conditions.

Each of mechanotransduction channels that we analyzed showed different functions under 3-D environments. For instance, DEG/ENaC MEC-4 regulates 3-D locomotion, whereas OSM-9 (TRPV) and TRP-4 (TRPN) mediate reorientation behaviors of the worm. Mutation in the glutamate receptor *glr-1*, which is involved specifically in the OSM-9-mediated mechanosensory pathway^26^, caused a phenotype similar to that of the *osm-9* mutants (Supplementary Fig. 6), implying the importance of the mechanosensation in the regulation of behaviors. TRP-4 channels play direct roles in proprioception mediated by the DVA neuron^24^ and in mechanotransduction mediated by a dopaminergic neuron CEP^11^,^27^. The rescue of the reduced reorientation phenotype of *trp-4* mutants by dopaminergic neuron-specific *trp-4* expression (Fig. 4e) suggests that the mechanosensation, not the proprioception, is involved in regulation of reorientation in 3-D environments. Further studies are needed to elucidate cellular and molecular mechanisms by which mechanosensory pathways regulate the worm behavior in 3-D conditions.

In 3-D environments, several mutant worms displayed various phenotypes that were not shown in 2-D conditions. This suggests that behavioral studies in 3-D environments are essential to understand complex functions of genetic and neural systems in animal behaviors. For this purpose, we believe that the 3DWT 2.0 can significantly contribute to the examination of various behaviors in 3-D natural conditions and afford new opportunities for understanding genetic and neural mechanisms underlying animal behaviors in natural 3-D environments.

## Methods

### Strains

*C. elegans* strains were maintained on NGM plates seeded with OP50 *E. coli* at 18˚C. After 3 days from hatching, young adult worms were examined for their locomotory behaviors. The following strains were analyzed in this study: N2 wild type, CB698 *vab-10(e698) I*, MT6318 *eat-4(n2474) III*, CB1339 *mec-4(e1339) X*, CB1515 *mec-10(e1515) X*, CX10 *osm-9(ky10) IV*, CX4544 *ocr-2(ak47) IV*, KP4 *glr-1(n2461)III*, RB1052 *trpa-1(ok999) IV* and TQ296 *trp-4 (sy695) I*. For *trp-4* rescue experiments, we used the following strains: TQ392 *trp-4(sy695); xuEx[Ptwk-16(DVA)::trp-4; Ptwk-16(DVA)::DsRed2b; Podr-1::RFP]* and TQ1716 *trp-4(sy695); xuEx584[Pdat-1::cTRP4(50* *ng)::sl2::yfp* *+* *Punc-122::GFP]*, which were gifts from X.Z.S Xu laboratory.

### Behavioral assays

To create a physical surrounding for behavioral assays, gelatin (G1890, Sigma Aldrich) was dissolved in M9 buffer at 50°C to a final concentration of 3% (w/v) and was briefly cooled down to 20°C. For experiments in two-dimensional (2-D) environments, a drop of gelatin solution was placed on a slide glass with two spacers (100 μm in thickness) (Supplementary Fig. 1a) and four worms were loaded into the droplet using a platinum wire worm pick. The droplet was pressed to a film by making a sandwich with another slide glass tightened with clamps and then solidified at 18°C. For experiments in three-dimensional (3-D) environments, quartz cuvettes (1.5 cm × 1.5 cm × 1.25 cm) were filled with the gelatin solution (Supplementary Fig. 1b), which were subsequently solidified at 18 °C. Ten worms were then inserted in the solidified gelatin by using a worm pick. For both 2-D and 3-D experiments, animals were habituated in the gelatin media for 30 minutes and four single worms were tracked for 3 minutes respectively. The culture and behavioral examination of worms were performed at 18 °C, because gelatin media were not sufficiently solidified at 20 °C.

### Vision hardware

PCO.1600 (14 bits; 1600 × 1200 pixels; PCO) camera 1 and PCO.edge (16 bits; 2560 × 2160 pixels; PCO) camera 2 with two identical objective lenses (×3 telecentric objective lens, Mitutoyo, NA 0.09, parfocal length 110 mm) were used for x- and y-view imaging and z-view imaging, respectively. The resolutions of the camera 1 and the camera 2 were 4 μm/pixel and 3 μm/pixel, respectively. The camera 2 received trigger signals from the camera 1 for synchronized imaging of x- and y-views (by camera 1) and z-views (by camera 2). The delay time for the triggered recording of z-views was less than 30 μs. For bright-field illumination, an LED (Precision LED Spotlights, Mightex systems) was used for each view.

### Tracker module

For automatic 3-D tracking, we programmed a tracker module, a custom software written in visual basic, which presents interfaces to adjust several parameters for real-time worm detection and motor control. ImagePro Plus (Media Cybernetics), which was also controlled by the module, was used to capture x- and y-views and to recognize worms in real-time using the parameters (Fig. 1b). Before initiating automatic 3-D tracking, a single worm was set near to the centers of x- and y-views, which correspond to the focal point of the imaging system, by manually controlling a motorized sample stage.

Next, a background image, taken with no gelatin cell, was subtracted from x- and y-views. The worm was then identified from the subtracted image based on two criteria (Fig. 1b): i) object intensity with less than 70% of the average intensity of the background image^7^ and ii) object size between 8000 μm^2^ ~ 80000 μm^2^. Afterwards, the centers of mass of the worm, calculated in one set of x- and y-views, were translated to the image centers by using the motorized stage (Fig. 1b). This automatic 3-D tracking was repeated with an interval of 1.4 s, which was required for real-time image analysis, motor positioning, and image data transfer. One set of three view images and the sample stage position were stored every time. In case of 2-D tracking, the same algorithm was applied to single images only from the x-view. All images were then binarized for quantitative analysis.

### Analyzer module

Reconstruction of 3-D trajectories and 3-D postures of worms were performed by using an analyzer module, which is a custom software written in MATLAB. The images from the z-view were first resized with a factor of 0.75 to adjust the resolution difference between the two cameras. To remove the spots in worms, morphological closing was applied to binary images^28^. Each binary image was then cropped into a box that fits the worm. The position of the box center in its original image was then taken as the worm position in each view. The worm positions in the x-and y-views were merged into a 3-D position (x, y, z). By compensating the 3-D position with the sample stage position, the worm position in the gelatin cube was estimated.

To reconstruct 3-D volume of a worm, we first generated back projection profiles of binary worm images from the cropped x-, y-, and z-images and aligned the centers of the three profiles to one point. The volume of a worm was defined as a group of voxels where all three profiles intersect.

To demonstrate traces of a worm, we obtained 3-D skeletons using 3-D thinning of the worm’s volumes^29^ and merged the skeletons into its trajectory. For the 2-D experimental data, the skeletonization of a worm was based on a two-subiteration thinning algorithm (algorithm 1 in ref. 30). To remove the branch noises in the skeleton, a skeleton of a worm was defined as the longest skeleton that connects two end points of the raw skeleton.

### Curving rate analysis

The histogram of curving rate showed two peaks near the forward direction (0°/frame) and reverse direction (180°/frame) (Supplementary Fig. 2). The first peak represents forward runs with gradual curving^17^. The other peak near 180° represents turning and reversals, which occur when worms reorient their directions^12^. Here, forward run was defined as the low curving rates less than 60°/frame whereas reorientation was defined as the high curving rates larger than 135°/frame. Less than 1% of the data exist within 60°–135°/frame.

### Non-planar deviation

To quantify 3-D postures, we first extracted the coordinates of the voxels that are included in each reconstructed volume of a worm. By using Principle Component Analysis (PCA), three principal axes (eigenvectors) and standard deviations (square roots of the eigenvalues) of the coordinates along the principal axes were calculated. The deviation values were normalized based on the sizes and the thicknesses of worms with the following calculations. First, the deviation to the third principal axis (R3) was divided by the deviation to the first axis (R1). Next, the ratio R3/R1 was subtracted with the least of the ratios of R3 to R1, which presumably originated from the worm thickness (Supplementary Fig. 3). The normalized deviation was called as non-planar deviation.

### Directional autocorrelation

To calculate directional autocorrelation during forward runs, velocity data sets of consecutive velocities for gradual curving (<60°/frame) was extracted. Unit direction vectors of the velocities were then calculated. The autocorrelation for a time lag τ is defined as below.
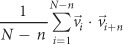


Here, N is the total number of the consecutive velocities of a set, n is the number of velocities recorded during the time lag, and 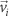 is the i^th^ unit direction vector.

## Author Contributions

N.K. designed the 3-D Worm Tracker 2.0, built the hardware and wrote the software. N.K., A.B.H., S.-J. V. L. and J.H.J designed the experiments. N.K. and A.B.H. conducted the experiments. N.K., A.B.H., Y.-J.Y., S.-J. V. L. and J.H.J analyzed data and wrote the manuscript.

## Additional Information

**How to cite this article**: Kwon, N. *et al*. Dissection of *C. elegans* behavioral genetics in 3-D environments. *Sci. Rep.* *5*, 9564; doi: 10.1038/srep09564 (2015).

## Supplementary Material

Supplementary Figures and Methods

Supplementary Movie 1

Supplementary Movie 2
