# Supplementary material for: Dissection of C. elegans behavioral genetics in 3-D environments
Source: Sci Rep. 2015 May 8;5:9564. doi: 10.1038/srep09564 (PMC4424945; doi:10.1038/srep09564)
Supplement: Supplementary Figures and Methods [file srep09564-s1.docx]

SUPPLEMENTARY INFORMATION

**Inauguration of 3-dimensional *C. elegans* behavioral genetics**

Namseop Kwon, Ara B. Hwang, Young-Jai You, Seung-Jae Lee and Jung Ho Je

| Supplementary Movie 1 | Three-dimensional (3-D) reconstructed images of a wild-type *C.elegans* recorded for 3 minutes. |
| --- | --- |
| Supplementary Movie 2 | A series of movies showing wild-type, *vab-10* and *eat-4* mutant animals that crawled in 3-D environments. |
| Supplementary Figure 1 | Two-dimensional (2-D) and three-dimensional (3-D) media for behavioral assays. |
| Supplementary Figure 2 | Curving rate distribution of wild-type animals in 2-D (7,841 frames) and 3-D (6,019 frames) environments. |
| Supplementary Figure 3 | Non planar deviation of worms in 2-D environments. |
| Supplementary Figure 4 | Distribution of non-planar deviation (NPD). |
| Supplementary Figure 5 | Trajectories of wild-type, *vab-10*, and *eat-4* mutant animals. |
| Supplementary Figure 6 | Locomotory behaviors of *ocr-2* and *glr-1* mutants. |

**Movie Legends**

**Supplementary Movie 1. Three-dimensional reconstructed images of a wild-type *C. elegans* recorded for 3 minutes.**

**Supplementary Movie 2.** **A series of** **movies showing wild-type, *vab-10* and *eat-4* mutant animals that crawled in 3-D environments.** The images were recorded with a fast frame rate of 13 frames/s, while manually tracking worms.

**Supplementary Figure 1**


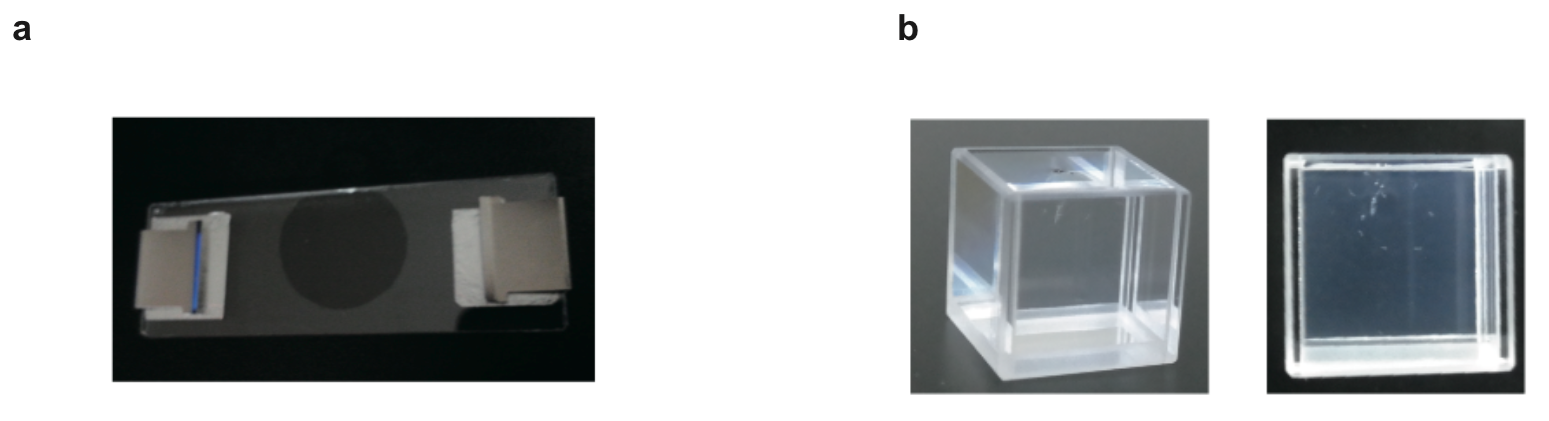


**Supplementary Figure 1.** **Two-dimensional (2-D) and three-dimensional (3-D) media for behavioral assays.** Gelatin-cover glass sandwich (<100 μm) (a) and gelatin filled square cuvettes (>2 cm^3^) (b) were used as 2-D and 3-D environments, respectively.

**Supplementary Figure 2**


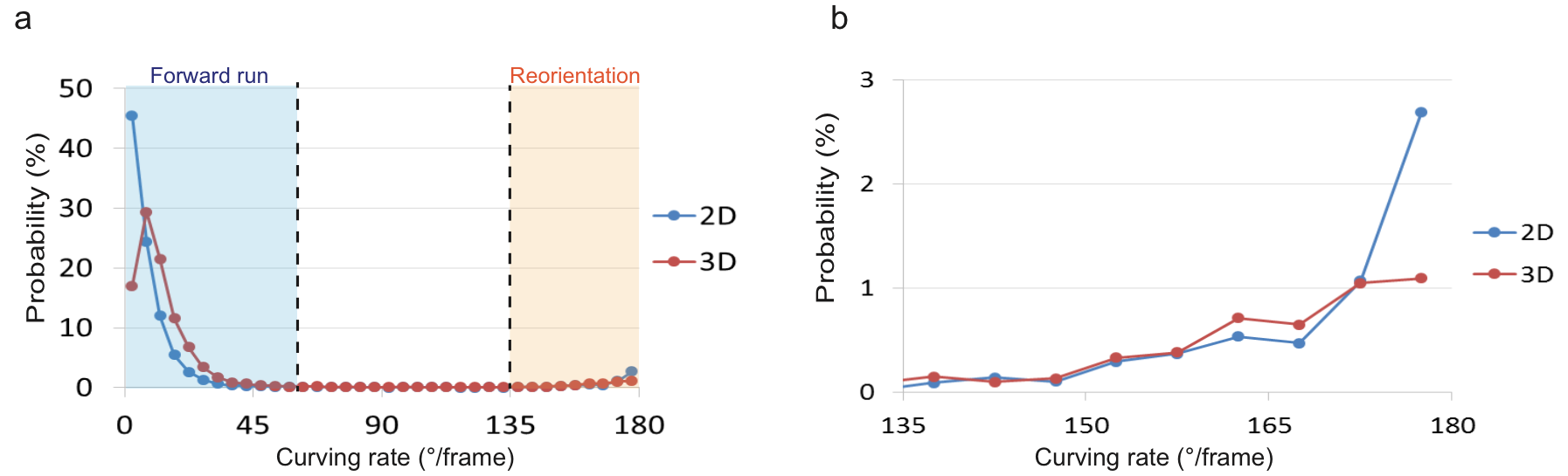


**Supplementary Figure 2. Curving rate distribution of wild-type animals in 2-D (7,841 frames) and 3-D (6,019 frames) environments.** (**a**) Both of the curving rate distributions from 2-D and 3-D experiments showed one large peak near 0° and one small peak near 180°, representing forward run and reorientation, respectively. (**b**) A magnified plot showing the small peaks in panel (**a**).

**Supplementary Figure 3**


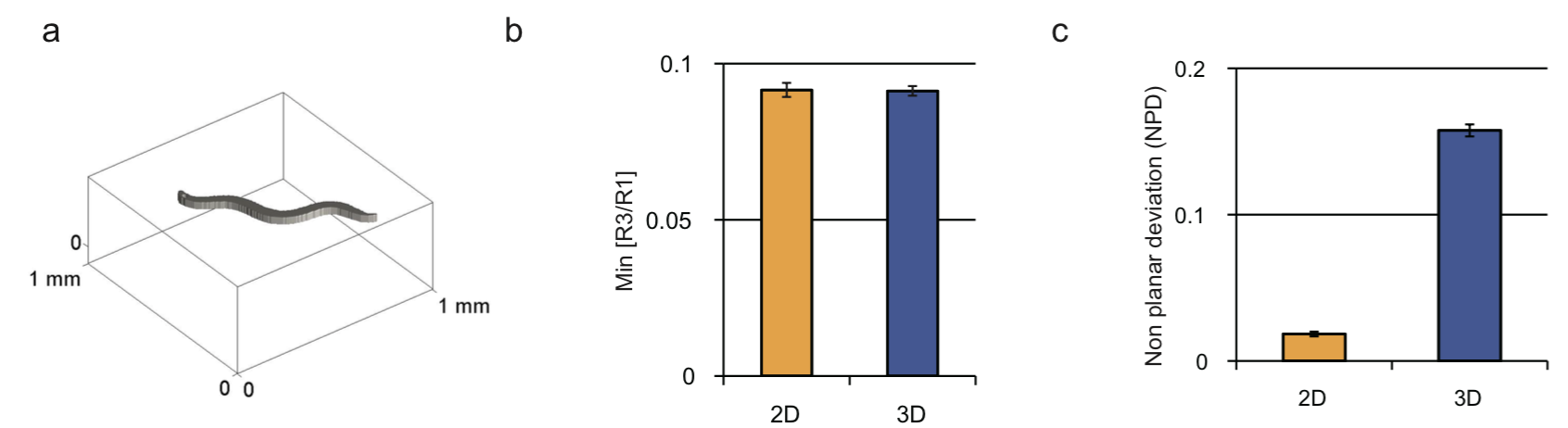


**Supplementary Figure 3. Non-planar deviation of worms in 2-D environments.** Worms crawling in 2-D environments were simulated by giving a thickness (60 μm, the average thickness measured from 15 worms) to 2-D images, as demonstrated in (**a**). (**b**) The least of the ratio of R3 to R1 obtained from the simulation of 2-D images and from the reconstructed 3-D images. The values from 2-D simulation are comparable to those from reconstructed 3-D images, indicating that the least values originated from the thicknesses of the worms. (c). Non-planar deviations (NPDs) of the worms in 2-D and 3-D conditions. The NPDs obtained from 3-D reconstruction are one order higher than those from 2-D simulation, suggesting that the NPDs represent an extra degree of freedom of movements in 3-D environments.

**Supplementary Figure 4**


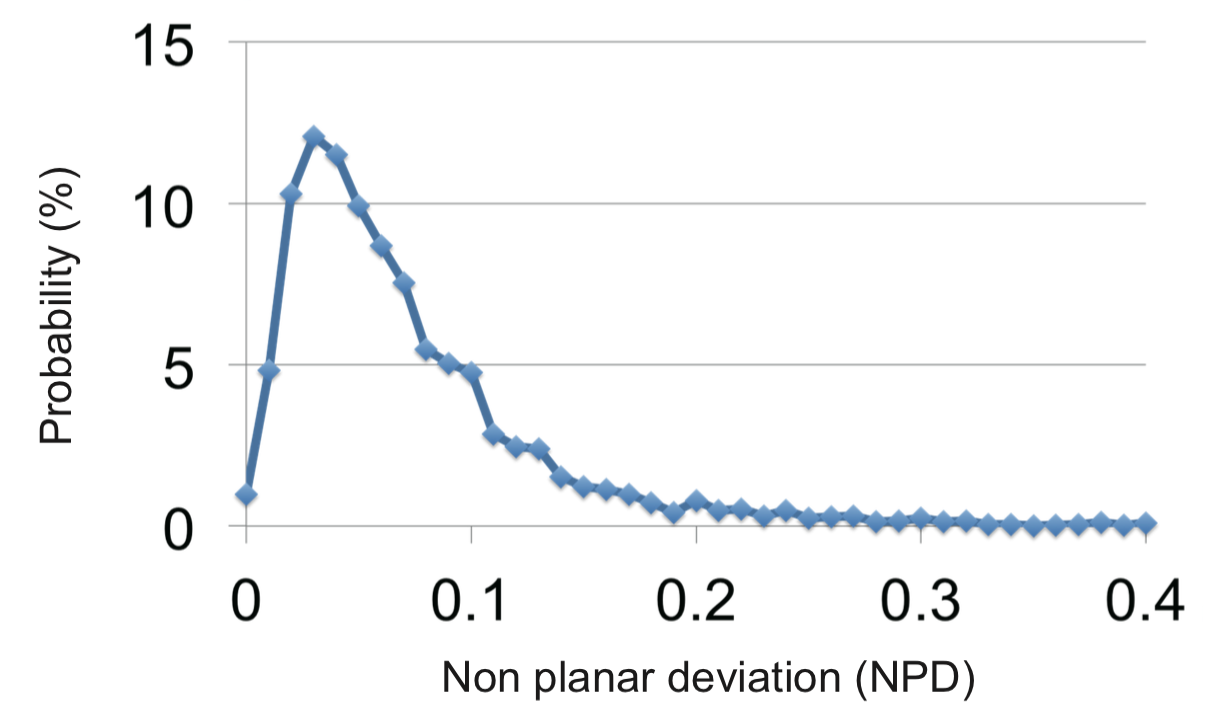


**Supplementary Figure 4. Distribution of non-planar deviations (NPDs).**  NPD distribution of wild-type worms moving in 3-D environments. A majority (99%) of the values were distributed between 0~0.3.

**Supplementary Figure 5.**


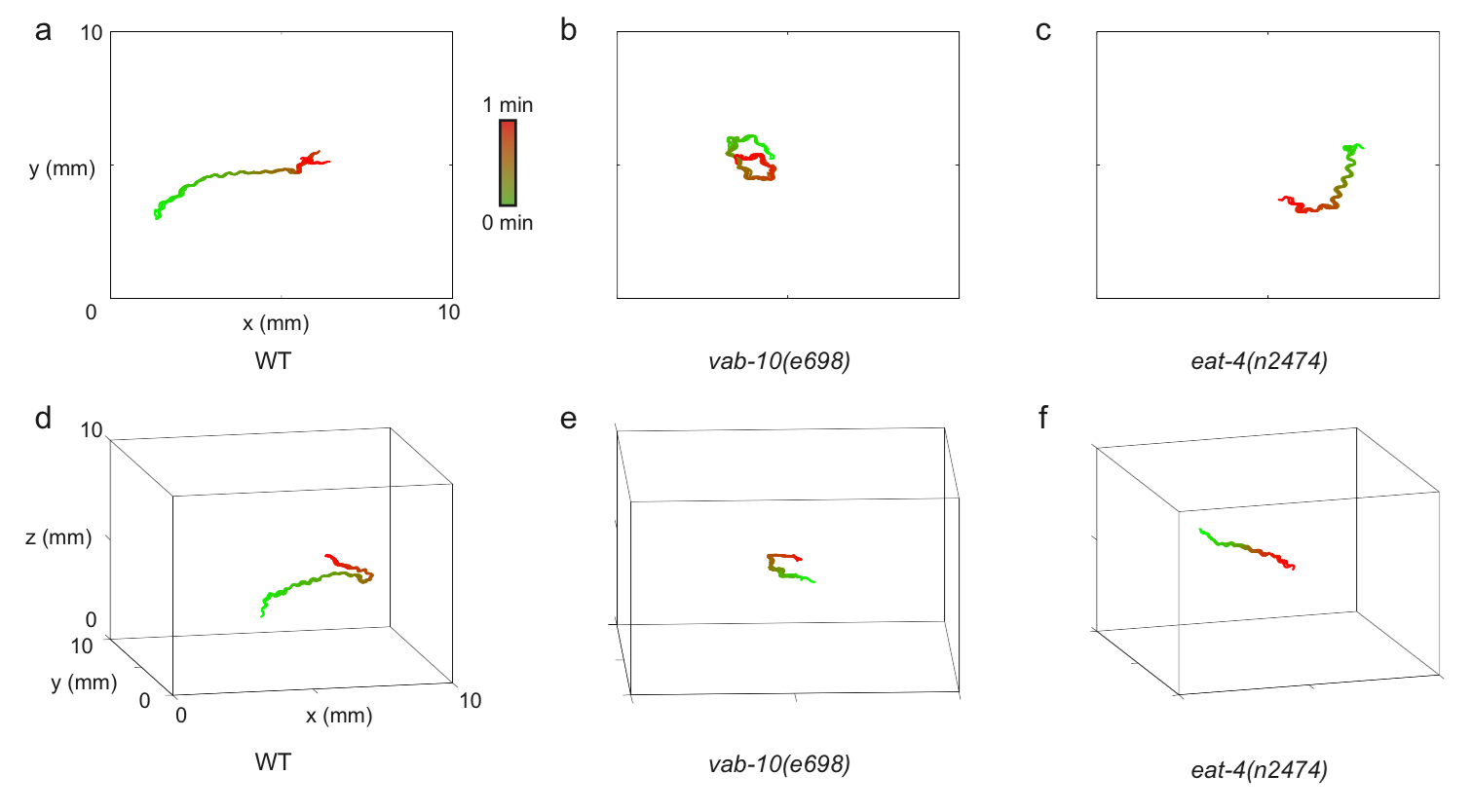


**Supplementary Figure 5. Trajectories of wild-type, *vab-10*, and *eat-4* mutant animals.** The representative trajectories of wild-type, *vab-10(e698)* and *eat-4(n2474)* animals in 2-D (**a-c**) and 3-D (**d-f**) environments for one minute. *vab-10* encodes two isoforms of spectraplakins, *vab-10A* and *vab-10B*, whose inhibition causes the detachment of cuticle from the muscles with abnormal forms of fibrous organelle and increases epidermal thickness, respectively^1^. Since the complete loss of *vab-10* leads to embryonic lethality, we used a hypomorphic mutant allele *e698* that contains a missense mutation in the *vab-10A* isoform, causing a minor head morphogenesis defect and immobile head^2^. *eat-4* encodes a homolog of a mammalian brain-specific Na^+^-dependent inorganic phosphate cotransporter 1 required for glutamate uptake and glutamatergic neurotransmission^3^.

**Supplementary Figure 6.**


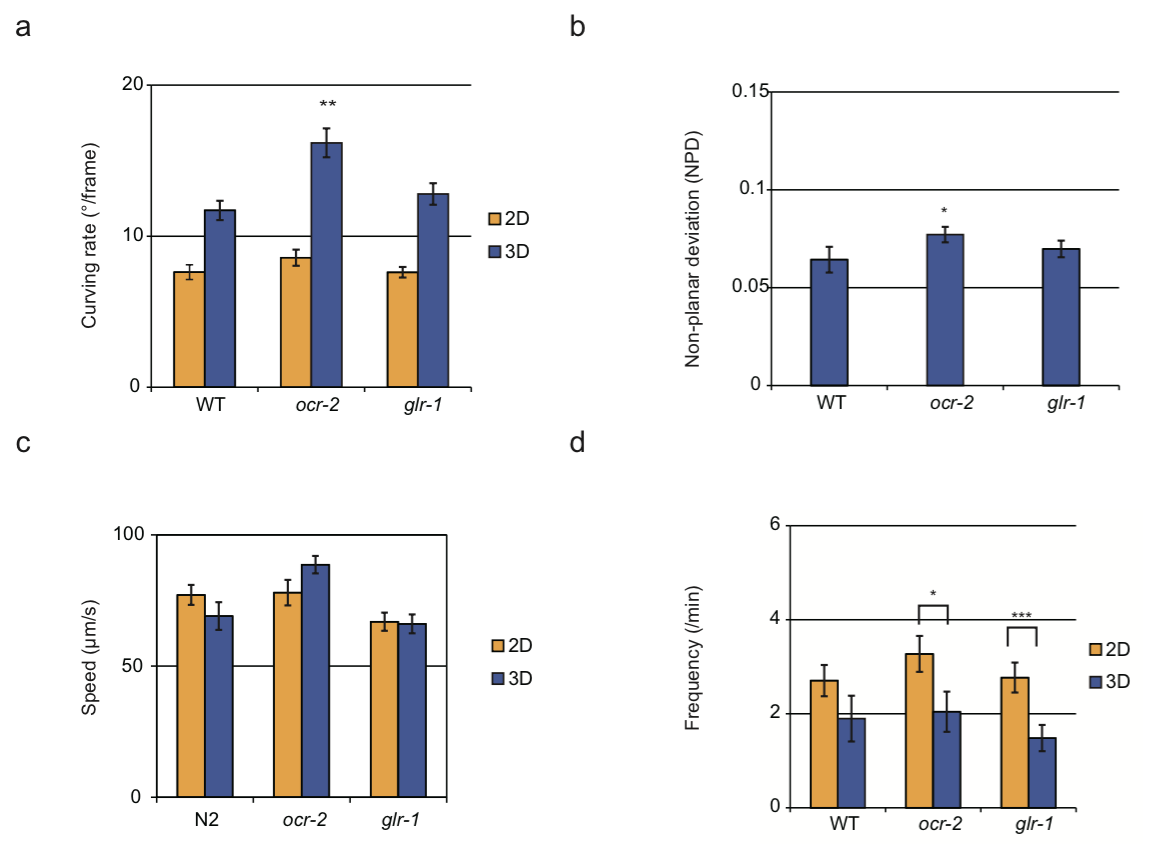


**Supplementary Figure 6. Locomotory behaviors of *ocr-2* and *glr-1* mutants.** (**a**-**b**) *ocr-2(ak47)* mutants showed increased curving rates for forward runs (**a**) and non-planar deviations (**b**) compared to wild-type animals. (**c-d**) *ocr-2(ak47)* and *glr-1(n2461)* mutants displayed comparable speeds in 2-D and 3-D conditions (**c**) but significant reduction in the reorientation frequencies under 3-D conditions compared with those in 2-D conditions (**d**) (n = 18 and 14 for wild type; n = 12 and 13 for *ocr-2*; and n = 26 and 29 for *glr-1* mutants in 2-D and 3-D conditions, respectively). Statistical analysis was performed to compare mutants with wild-type animals (**a-b**) or between 2-D and 3-D data (**c-e**). Error bars represent SEM (**P <0.005*, ***P<0.001*, ****P<0.0005*, Mann-Whitney U-test).

**References**

1. Bosher, J. M. *et al*. The *Caenorhabditis elegans* *vab-10* spectraplakin isoforms protect the epidermis against internal and external forces. *J. Cell. Biol.* **161**, 757–768 (2003).
2. Hodgkin, J. Male phenotypes and mating efficiency in *Caenorhabditis elegans*. *Genetics* **103**, 43–64 (1983).
3. Lee, R. Y. N., Sawin, E. R., Chalfie, M., Horvitz, H. R. & Avery, L. EAT-4, a homolog of a mammalian sodium-dependent inorganic phosphate cotransporter, is necessary for glutamatergic neurotransmission in caenorhabditis elegans. *J. Neurosci.* **19**, 159–167 (1998).
